# Pulmonary Coccidioidomycosis Linked to Shipping Containers, Taiwan, 2025

**DOI:** 10.3201/eid3209.260714

**Published:** 2026-09

**Authors:** Allen C. Huang, Tsung-Han Lee, Min-Nan Hung, Mei-Hui Liao, Hsueh-Mei Chiang, Min-Hua Hsieh, Tein-Yao Chang, Shu-Min Lin

**Affiliations:** Chang Gung University Graduate Institute of Clinical Medical Sciences, Taoyuan, Taiwan (A.C. Huang); St. Paul's Hospital, Taoyuan (A.C. Huang, S.-M. Lin); Linkou Chang Gung Memorial Hospital, Taoyuan (A.C. Huang, S.-M. Lin); Taiwan Centers for Disease Control, Taipei, Taiwan (T.-H. Lee, M.-N. Hung, M.-H. Liao, H.-M. Chiang, M.-H. Hsieh); National Defense Medical University Institute of Preventive Medicine, Taipei (T.-Y. Chang); National Defense Medical University Graduate Institute of Biodefense, Taipei (T.-Y. Chang); Chang Gung University School of Medicine, Taoyuan (S.-M. Lin)

**Keywords:** Coccidioidomycosis, coccidioides, environmental exposure, occupational exposure, disease transmission, infectious, spores, fungi, Taiwan

## Abstract

A shipping container yard worker from Taiwan contracted laboratory-confirmed pulmonary coccidioidomycosis. He reported no travel history to endemic regions. Occupational exposure to aerosolized dust or soil residues on maritime shipping containers from endemic areas is likely. Our findings highlight international shipping as a potential pathway for introducing geographically restricted pathogens into nonendemic regions.

Coccidioidomycosis is caused by inhaling fungal spores produced by the dimorphic fungi *Coccidioides immitis* and *C. posadasii*, which inhabit arid soil primarily in the southwestern United States, northern Mexico, and parts of Central and South America (*1*). Outside those endemic areas, most cases are associated with travel or prior residence (*2*). Transmission by contaminated materials or objects from endemic regions is rare (*2*–*4*). However, increasing cargo movement from endemic regions raises the possibility that contaminated dust or soil residues might be transported and infect persons living in nonendemic regions.

In Taiwan, previously reported cases of coccidioidomycosis have been identified exclusively among persons with exposure to endemic areas (*5*). We report a case of locally acquired pulmonary coccidioidomycosis in a patient who had never traveled to the Americas. The patient provided written informed consent for the publication of his clinical history, occupational data, and relevant images.

The patient, a man in his 50s, was a smoker with no known underlying conditions. In August 2025, he sought care for a 2-week history of cough, fatigue, and intermittent dizziness. Chest radiography revealed a mass in the right upper lobe (RUL) with right hilar lymphadenopathy. Chest computed tomography (CT) revealed an RUL 3.0 × 2.0 cm solid mass touching the pleura, as well as enlarged right hilar, mediastinal lymph nodes, and multiple pulmonary nodules bilaterally (Figure 1). Because of the patient’s age and smoking history, clinicians suspected a primary RUL lung malignancy with mediastinal and intrapulmonary metastases. The patient’s serum tumor markers, including cancer antigen 19–9, carcinoembryonic antigen, squamous cell carcinoma antigen, and cancer antigen 125, were within reference ranges.

**Figure 1:**
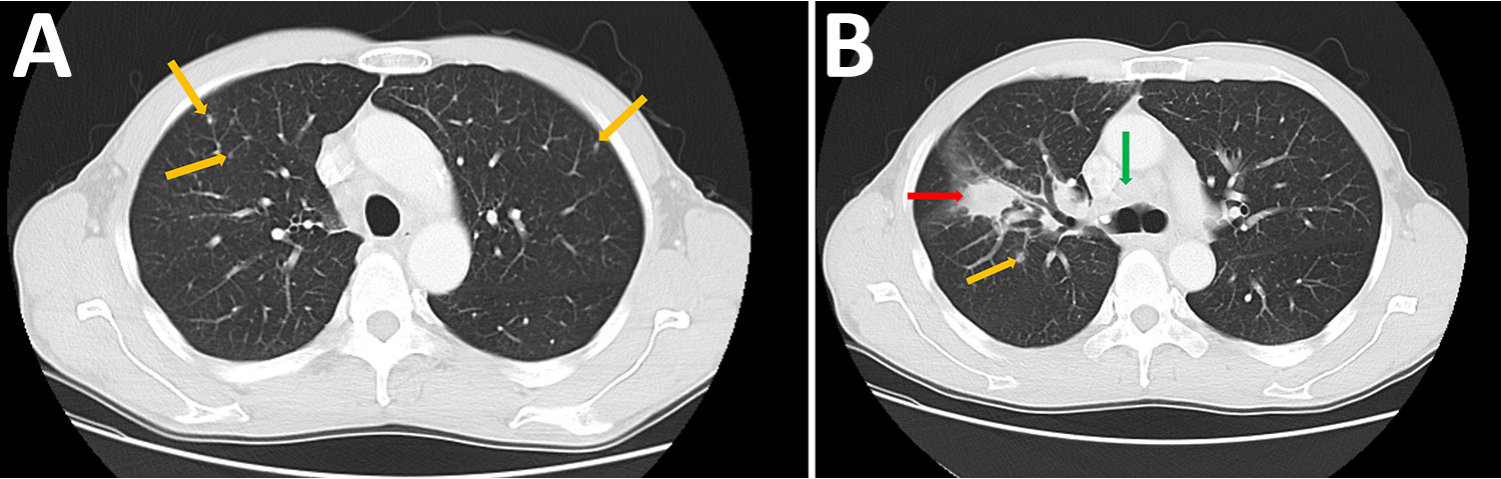
Chest computed tomography findings in a maritime container yard worker with pulmonary coccidioidomycosis, Taiwan, 2025. A) Axial lung-window images reveal multiple bilateral pulmonary nodules (orange arrows). B) A 3.0 × 2.0 cm solid mass is visible in the right upper lobe (red arrow), and right hilar lymph nodes are enlarged (green arrow).

A flexible bronchoscopy retrieved tissue for histopathologic and microbiological evaluation. Endobronchial ultrasound did not show the pulmonary mass, precluding transbronchial biopsy. Pathologic examination of the fine-needle aspirate from the right hilar lymph node found granulomatous inflammation but no evidence of malignancy. Sputum and bronchoalveolar lavage specimens were negative for *Mycobacterium tuberculosis* by using PCR and culture. Fungal culture of bronchoalveolar lavage fluid yielded a colony morphologically consistent with *Coccidioides* spp. Serologic lateral flow assay was positive for *Coccidioides* antibodies, and enzyme immunoassay confirmed reactivity for both IgM and IgG. Because of potential serologic cross-reactivity among endemic dimorphic fungi in Taiwan, molecular testing by internal transcribed spacer sequencing and the CocciDx (Translational Genomics Research Institute, https://www.tgen.org) real-time PCR was performed, confirming *C. immitis* (*6*). The patient was treated with fluconazole and had gradual clinical and radiologic improvement.

The patient reported no travel to coccidioidomycosis-endemic regions nor indirect exposure in the month before illness onset, including contact with items brought by visitors from endemic areas or personally sourced from these regions. The patient had worked for years at a maritime container yard where shipping containers were stored, repaired, and cleaned after cargo removal. His duties included hammering deformed container panels and cleaning container surfaces by using high-pressure water jets, both performed without respiratory protection. Those activities could disturb residual soil or dust on container surfaces, aerosolizing *Coccidioides* arthroconidia spores and making them easier to inhale (Figure 2).

**Figure 2:**
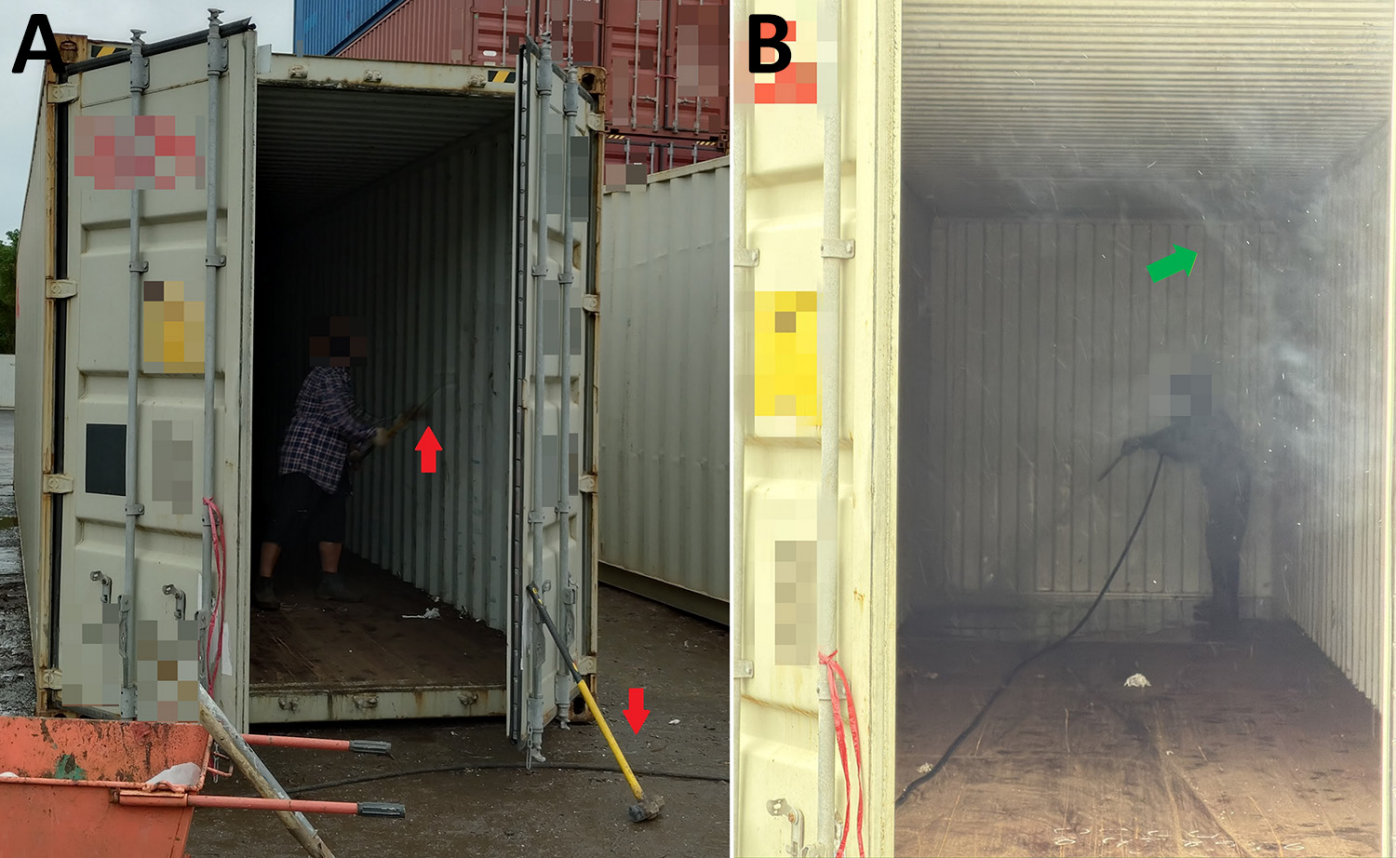
Occupational activities at a maritime container yard in Taiwan, 2025, that might generate airborne *Coccidioides immitis* from soil or dust. A) Red arrows indicate hammers used for hammering of deformed container panels during metal surface repair. B) Green arrow indicates backsplash from high-pressure water jet cleaning of interior container surfaces. Images do not depict the patient reported in this case or any specific containers he worked on.

Although the specific containers involved could not be identified, the containers the patient might have worked in were associated with ports in California and Washington, USA. Because *C. immitis* is native to parts of California, Baja California, and eastern Washington (*1*), we considered the possibility of exposure to contaminated soil or dust transported in shipping containers. Our conclusion that this pathway was possible is supported by a previous report suggesting *Coccidioides* arthroconidia could be transported with shipped goods and aerosolized during container sweeping (*4*). It is also biologically plausible because of the documented environmental persistence and fomite stability of *Coccidioides* arthroconidia (*7*,*8*). Together, the absence of travel history and routine aerosol-generating container work support occupational exposure as the most likely route of acquisition.

The first limitation of our investigation is the omission of container surface environmental sampling, which precluded direct detection of aerosolized arthroconidia and prevented definitive source attribution. Second, the lack of serologic assessment among exposed co-workers limited our ability to evaluate potential shared occupational exposure. Finally, without prior serologic or clinical data, we could not distinguish recent infection from reactivation, although we considered reactivation less likely because of the absence of recognized risk factors (*9*,*10*).

With continued global commerce integration, traditional geographic boundaries of infectious diseases might be less distinct. In nonendemic regions, early diagnosis requires clinicians to consider coccidioidomycosis in patients with compatible clinical and radiologic findings and to ask about occupational exposure to imported dust- or soil-contaminated materials, including maritime containers, especially when travel history to endemic areas can be ruled out. For occupational prevention, control measures should follow the hierarchy of controls and include standardized protocols to minimize aerosolization during container maintenance, worker education and training, periodic health monitoring, and the use of fit-tested respiratory protection during high-risk activities. Surveillance in international shipping settings also might help identify exposure risks and inform strategies to prevent this emerging, potentially underrecognized occupational hazard.

## References

[1] Kollath DR, Miller KJ, Barker BM. The mysterious desert dwellers: *Coccidioides immitis* and *Coccidioides posadasii*, causative fungal agents of coccidioidomycosis. Virulence. 2019;10:222–33. 10.1080/21505594.2019.1589363 30898028 PMC6527015

[2] Desai SA, Minai OA, Gordon SM, O’Neil B, Wiedemann HP, Arroliga AC. Coccidioidomycosis in non-endemic areas: a case series. Respir Med. 2001;95:305–9. 10.1053/rmed.2000.1039 11316114

[3] Stagliano D, Epstein J, Hickey P. Fomite-transmitted coccidioidomycosis in an immunocompromised child. Pediatr Infect Dis J. 2007;26:454–6. 10.1097/01.inf.0000259231.95285.bc 17468663

[4] Tang THC, Tsang OTY. Images in clinical medicine. Fungal infection from sweeping in the wrong place. N Engl J Med. 2011;364:e3. 10.1056/NEJMicm1007348 21226571

[5] Li Y-C, Lin M-W, Wu U-I, Lu C-W, Chen P-H, Tsai T-M, et al. Coccidioidomycosis: an emerging disease in Taiwan. J Formos Med Assoc. 2026;S0929-6646(26)00005-7. 41513536 10.1016/j.jfma.2026.01.005

[6] Litvintseva AP, Marsden-Haug N, Hurst S, Hill H, Gade L, Driebe EM, et al. Valley fever: finding new places for an old disease: *Coccidioides immitis* found in Washington State soil associated with recent human infection. Clin Infect Dis. 2015;60:e1–3. 10.1093/cid/ciu681 25165087 PMC4296125

[7] Chow NA, Kangiser D, Gade L, McCotter OZ, Hurst S, Salamone A, et al. Factors influencing distribution of *Coccidioides immitis* in soil, Washington state, 2016. MSphere. 2021;6:e0059821. 10.1128/mSphere.00598-21 34730378 PMC8565518

[8] Stevens DA, Clemons KV, Levine HB, Pappagianis D, Baron EJ, Hamilton JR, et al. Expert opinion: what to do when there is *Coccidioides* exposure in a laboratory. Clin Infect Dis. 2009;49:919–23. 10.1086/605441 19663562

[9] Bhardwaj S, Tewari A, Tewari R, Kogan S. The fungus among us: latency, reactivation, and the expanding clinical spectrum of coccidioidomycosis. Cureus. 2026;18:e107243. 10.7759/cureus.107243 42153078 PMC13180437

[10] Crum NF. Coccidioidomycosis: a contemporary review. Infect Dis Ther. 2022;11:713–42. 10.1007/s40121-022-00606-y 35233706 PMC8887663

